# Early caplacizumab and obinutuzumab enable successful treatment of relapsing thrombotic thrombocytopenic purpura without therapeutic plasma exchange: a case report

**DOI:** 10.3389/fimmu.2025.1588471

**Published:** 2025-04-28

**Authors:** Judith Schimpf, Patrizia Haller, Emanuel Zitt

**Affiliations:** ^1^ Department of Internal Medicine 3 (Nephrology, Dialysis and Hypertension), Landeskrankenhaus (LKH) Feldkirch, Feldkirch, Austria; ^2^ Vorarlberg Institute for Vascular Investigation and Treatment (VIVIT), Feldkirch, Austria; ^3^ Agency for Preventive and Social Medicine (aks), Bregenz, Austria

**Keywords:** caplacizumab, obinutuzumab, thrombotic microangiopathy, therapeutic plasma exchange, thrombotic thrombocytopenic purpura

## Abstract

Thrombotic thrombocytopenic purpura (TTP) is a rare and life-threatening disorder due to a severe acquired or inherited ADAMTS13 deficiency. So far, therapeutic algorithms almost universally include the prompt initiation of therapeutic plasma exchange (TPE). We firstly report a 55-year-old female with a history of relapsing TTP who was managed exclusively with caplacizumab, steroids and the second generation fully humanized anti-CD20 monoclonal antibody obinutuzumab during a relapse without the need of TPE throughout the whole disease course. This case illustrates the safety and effectiveness of a TPE-free TTP management using prompt initiation of caplacizumab and obinutuzumab.

## Introduction

Thrombotic thrombocytopenic Purpura (TTP) is a rare, potentially life-threatening thrombotic microangiopathy characterized by microangiopathic hemolytic anemia, severe thrombocytopenia, and organ ischemia due to disseminated microvascular platelet-rich thrombi. It is caused by a severe deficiency (activity <10%) in ADAMTS13 (a disintegrin and metalloproteinase with thrombospondin type 1 motif, member 13), a protease responsible for cleaving von Willebrand factor (vWF), mainly due to autoantibodies against ADAMTS13 (iTTP), but a rare inherited form (cTTP) is also known. The clinical course is characterized by a relapsing tendency after the initial episode, which mostly presents during adulthood (1). This disease requires prompt treatment, with therapeutic plasma exchange (TPE) being the cornerstone of standard therapy algorithms, as indicated by recent guidelines (2).

Caplacizumab, a nanobody that binds to the A1 domain of vWF, thereby blocking VWF-platelet interactions, became the first regulatory-approved therapy for iTTP after showing clinical benefits and an acceptable safety profile in the HERCULES trial (3). Caplacizumab was approved in combination with daily plasma exchange until normalization of platelet counts.

Early suspicion with known TTP in relapse might enable a TPE-free treatment with rapid initiation of caplacizumab therapy. Here, we describe our first patient with relapsing TTP who was successfully treated without TPE using prompt initiation of caplacizumab and the second generation fully humanized anti-CD20 monoclonal antibody obinutuzumab.

## Case report

A 55-year-old, female patient presented to our emergency department with bruises, fatigue and headaches. She had no recollection of trauma but reported visual disturbances over the past few weeks. Her medical history included relapsing TTP, which was first diagnosed in 2009. Upon initial diagnosis, there was extensive organ involvement, including the central nervous system, heart, lungs, liver, kidneys, spleen, intestines, and stomach, indicating a severe systemic form of the disease. In 2009, the patient also experienced the first two relapses, which were treated with a total of 41 sessions of plasma exchange, vincristine (3 x 1 mg), rituximab (2 x 1 g), and oral steroid therapy.

In January 2015, the patient had another relapse of TTP, triggered by autoimmune hyperthyroidism, which was treated with 11 plasma exchange sessions and Rituximab (1500 mg in total).

In October 2024 she presented again in the evening hours with the symptoms described above. Laboratory results showed thrombocytopenia with 67 G/L (normal range 150–450 G/L), suggesting a relapse of TTP. Consistent with this, schistozytes (12 ‰) were detected as sign of hemolysis. Other typical hemolysis parameters (LDH, bilirubin, hemoglobin) were within normal limits. Kidney function was normal, but urinalysis revealed glomerular hematuria with dysmorphic erythrocytes and mild albuminuria with an urinary albumin/creatinine ratio of 167 mg/g. The calculated PLASMIC score of 4 points (no active cancer, no history of solid organ transplantation, INR <1.5, creatinine < 2.0mg/dL) indicated low risk (https://www.mdcalc.com/calc/10200/plasmic-score-ttp), in the French Mortality in TTP Score (MITS) the patient had 0 points indicating a low in-hospital mortality of 0.72% (https://practical-haemostasis.com/Clinical%20Prediction%20Scores/Formulae%20code%20and%20formulae/Formulae/TMAs/French_MITS_score.html). We promptly initiated high-dose corticosteroid treatment (250 mg prednisolone iv on day 1, followed by 80mg orally on the second day) and administered caplacizumab (10 mg, first dose intravenously). In the short-term follow-up after 6 hours we observed no further decrease but a mild increase in platelet count (71 G/L), allowing us to continue the initially chosen treatment approach avoiding TPE after shared decision-making with the patient. The next day, the ADAMTS-13 activity test results were available, showing a significantly reduced activity of 4% (normal activity: 60-121%, modified Bethesda Assay), with no inhibitor detected (as was also the case during her prior episodes). Because of her known rituximab intolerance, which presented as serum sickness disease after rituximab application during her last relapse in 2015, the fully humanized second generation monoclonal anti-CD20 antibody obinutuzumab (1000 mg) was administered at the third day. Treatment with caplacizumab was continued daily subcutaneously during her stay in the inpatient ward which lasted for a total of 8 days. Platelet count normalized on the fourth day.

**Figure 1:**
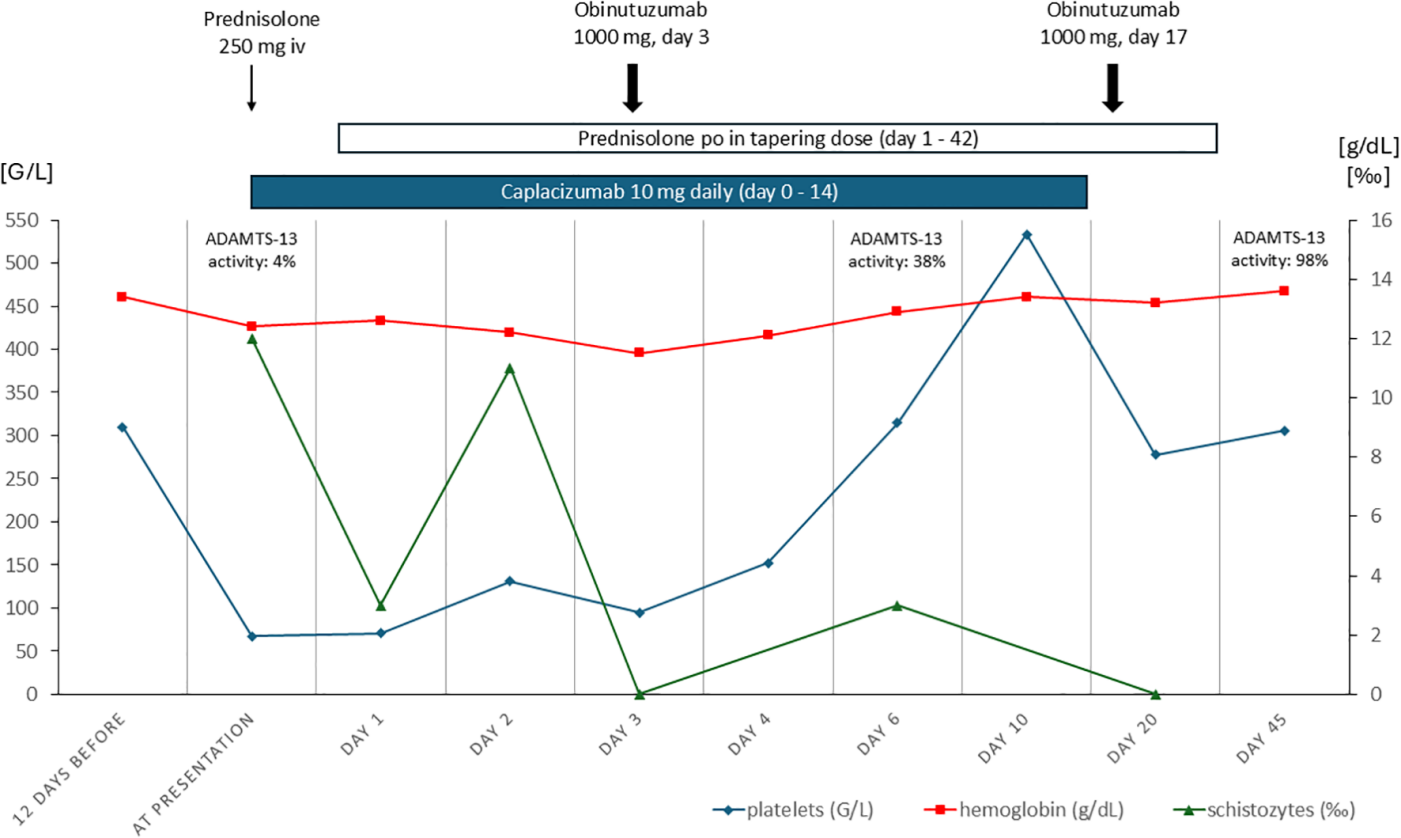
Laboratory and therapeutic time course.

Over the course of her treatment, the platelet counts steadily improved, and the patient remained hemodynamically stable without new symptoms. Six days later, the ADAMTS-13 activity had significantly improved to 38%. On the following day she was discharged in good general condition. Caplacizumab was administered subcutaneously for a total of 14 days and was stopped after repeated demonstration of normalized platelet count and ADAMTS13 activity (ADAMTS-13 activity of 86% 13 days after first obinutuzumab). The second dose of obinutuzumab (1000 mg) was repeated two weeks after the first dose. Neither caplacizumab nor obinutuzumab caused any side effects, especially no signs of bleeding, infection or infusion-related adverse events. Corticosteroid therapy was tapered on an outpatient basis and was stopped after six weeks (**Figure 1**). During regular follow-up visits no recurrence was noted, glomerular hematuria and albuminuria resolved completely and the patient remained asymptomatic.

## Discussion

Thrombotic thrombocytopenic purpura is a serious life-threatening medical emergency that requires immediate treatment. This patient has had a complex course of TTP with multiple relapses over a 15-year period. Our case demonstrates that, in certain circumstances, the condition can be managed without the standard treatment including TPE.

During the recent TTP relapse no inhibitor could be detected in our patient using the modified Bethesda assay, as was the case during her earlier episodes in 2015 and 2009. Negative inhibitor results can occur in some cases of early iTTP or be false negative in the case of immunoglobulin subtypes that are not detected by the immunoassay used. False-negative autoantibody testing can also occur with low-antibody titers or if autoantibodies are highly bound in antigen–antibody complexes (4).

Caplacizumab was tested and approved for use in iTTP together with TPE (3). This combined therapeutic approach is reasonable especially in patients presenting with thrombotic microangiopathy and suspected iTTP for the first time. Nevertheless, TPE being the cornerstone of TTP therapy until now is an invasive procedure, and complications might arise from vascular access, plasma side effects and its additional immunosuppressive effect. Additionally, it requires logistical and technical effort. Therefore, TPE-free TTP management would be beneficial to patients and care givers. A known history of TTP enables the early application of caplacizumab in the event of relapse, particularly when the patient is stable and there are no acute life-threatening complications. In such a situation a TPE-free therapeutic algorithm can be applied after shared decision making with the informed patient.

Kühne et al. (5) provided evidence in their retrospective multi-center cohort study of a possible TPE-free treatment approach. Treatment with caplacizumab and immunosuppression without TPE was successful in 90.5% of their patients. There were no significant differences between the non-TPE group and the TPE control group regarding time to platelet count normalization (3 vs. 4 days), clinical response (97.6% vs. 96.6%), relapses or mortality. Compared to a median caplacizumab treatment duration of 21.5 days in their cohort, we successfully applied a shorter duration of 14 days in our protocol.

The second-generation type II fully humanized monoclonal anti-CD20 antibody obinutuzumab exhibits a stronger B-cell depletion than its first-generation analogue rituximab due to different cytotoxicity. Whereas rituximab induces complement-dependent cytotoxicity, moderate antibody-dependent cellular cytotoxicity and promotes caspase-dependent apoptosis, obinutuzumab does not induce significant complement-dependent cytotoxicity, but instead relies on antibody-dependent cellular cytotoxicity, antibody-dependent cellular phagocytosis and direct cell death (6, 7). The glycoengineered and modified Fc region of obinutuzumab contributes to the improved B-cell depleting capacity (8). Obinutuzumab might therefore enable a faster reduction of causal autoantibodies in iTTP allowing for a TPE-free management. First registry data from France support this assumption. Weisinger et al. recently reported about the successful and safe obinutuzumab treatment of iTTP-patients with rituximab-resistance or intolerance (9). From 2020 to 2024 a total of 60 patients, all of whom had received rituximab either in the acute phase or during preemptive treatment before obinutuzumab, were treated with a median of 3 (IQR 1-4) obinutuzumab infusions. 85% of patients showed improved ADAMTS-13 activity ≥20%, with 72% of all patients reaching a complete response (ADAMTS-13 activity ≥50%). Patients who did not respond to prior rituximab therapy were less likely to achieve ADAMTS-13 improvement (≥20%) compared to patients intolerant to rituximab (27/35 (77%) vs 27/28 (96%), p<0.05). The median time of 76 days (IQR 33-234) to normalization of ADAMTS-13 activity was much longer in this study compared to our case (13 days). The French report did not mention concomitant treatment with caplacizumab, which might explain this difference.

From the patient´s perspective, the TPE-free management was highly welcome after complete treatment, because the patient was well informed about vascular access, the procedure of TPE and its associated risks and challenges due to her medical history and former experiences during prior relapses. Although the patient initially hesitated to proceed with a TPE-free management – as TPE was always designated as life-saving therapy during initial disease presentation and earlier relapses -, she finally agreed after in-depth information and with the prospect of avoiding TPE.

In conclusion, our case report supports the successful application of a TPE-free therapy algorithm with relapsing TTP using caplacizumab, steroids and obinutuzumab. This approach might be limited to patients with known TTP in the event of an early relapse but should be evaluated in future prospective randomized studies in patients with initial diagnosis of TTP. There are two ongoing studies using caplacizumab (NCT05468320) and recombinant ADAMTS13 (NCT05714969) initially without plasma exchange, in acute iTTP to determine whether plasma exchange can be safely omitted.

## Data Availability

The original contributions presented in the study are included in the article/supplementary material. Further inquiries can be directed to the corresponding author.
